# Patch testing in symmetrical drug-related intertriginous and flexural exanthema (sdrife)

**DOI:** 10.1186/2045-7022-4-S3-P76

**Published:** 2014-07-18

**Authors:** Razvigor Darlenski

**Affiliations:** 1 Tokuda Hospital Sofia, Bulgaria

## 

Herein we present a case of a 38-year-old female Caucasian who developed exanthema four days after surgical dental intervention. Skin symptoms included asymptomatic confluent erythematous papules and macules on the buttocks, lateral aspects of the abdomen, and axillas. After the dental surgery she was prescribed clindamycin for 7 days as a routine antibiotic prophylaxis. The patient had suffered from similar complaints upon previous intake of antibiotics. She was in otherwise general good health with no further complaints. Upon admittance, clidnamycin was stopped and symptomatic treatment with 40mg/day methylprednisolone was initiated, with tapering the dose within the next 7 days. No laboratory abnormalities were documented in the blood and urine analysis, liver, kidney and pulmonary function. The patients improved in a week with complete resolution of symptoms within 10 days. Six weeks after resolution of skin symptoms patch test was performed with European standard series and antibiotic prepared in our laboratory. Patch test result revealed positive reactions to nickel sulfate and clindamycin (10% in petrolatum). No positive reaction to the antibiotic was witnessed in the following testing of 5 healthy volunteers. Detailed discussion revealed that our patient has used topical clindamycin vaginal globules in the past without history of any topical or systemic reaction at the time. Upon the case history, the clinical picture, and the patch testing we set the diagnosis of Baboon syndrome also known as symmetrical drug-related intertriginous and flexural exanthema (SDRIFE). We discuss the need for patch testing the possible etiological agents and the pathophysiological mechanism of this drug reaction.

